# Drivers of change in weight‐for‐height among children under 5 years of age in Ethiopia: Risk factors and data gaps to identify risk factors

**DOI:** 10.1111/mcn.13392

**Published:** 2022-06-20

**Authors:** Meron Girma, Alemayehu Hussein, Kaleab Baye, Aregash Samuel, Cornelia van Zyl, Masresha Tessema, Stanley Chitekwe, Arnaud Laillou

**Affiliations:** ^1^ National Information Platforms for Nutrition (NIPN) Ethiopian Public Health Institute Addis Ababa Ethiopia; ^2^ Center for Food Science and Nutrition Addis Ababa University Addis Ababa Ethiopia; ^3^ National Information Platforms for Nutrition (NIPN) International Food Policy Research Institute Addis Ababa Ethiopia; ^4^ United Nations Children's Fund (UNICEF) Addis Ababa Ethiopia

**Keywords:** acute malnutrition, Ethiopia, prevention, risk factors, wasting, weight for height

## Abstract

The prevention of wasting should be a public health priority as the global burden of acute malnutrition is still high. Gaps still exist in our understanding of context‐specific risk factors and interventions that can be implemented to prevent acute malnutrition. We used data from the four rounds of the Ethiopia Demographic and Health Survey (2000–2016) to identify risk factors that have contributed to the change in weight‐for‐height *z*‐score (WHZ) among children under 5 years of age. We performed a pooled linear regression analysis followed by a decomposition analysis to identify relevant risk factors and their relative contribution to the change in WHZ. Modest improvements in WHZ were seen between 2000 and 2016. The sharpest decrease in mean WHZ occurred from birth to 6 months of age. Perceived low weight at birth and recent diarrhoea predicted a decline in WHZ among children aged 0–5, 6–23 and 23–59 months. Less than 50% of the change in WHZ was accounted for by the change in risk factors included in our regression decomposition analysis. This finding highlights data gaps to identify context‐specific wasting risk factors. The decline in the prevalence of recent diarrhoea (15% of the improvement), decline in low birth size (7%–9%), and an increase in wealth (15%–30%) were the main risk factors that accounted for the explained change in WHZ. Our findings emphasize the importance of interventions to reduce low birthweight, diarrhoea and interventions that address income inequities to prevent acute malnutrition.

## Key messages

- The sharpest decrease in weight‐for‐height *z*‐score (WHZ) was seen from birth to 6 months of age.
- Of the factors included in this analysis, perceived low size at birth and recent diarrhoea were the main drivers of change in WHZ.
- More than 50% of the improvement in WHZ was unexplained highlighting data gaps to understand context‐specific risk factors.

## Graphical abstract

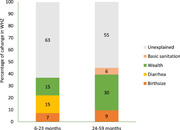

## 1 INTRODUCTION

Globally approximately 47 million children under 5 years of age suffer from acute malnutrition (United Nations Children's Fund [UNICEF], World Health Organization [WHO] et al., 2020). Furthermore, acute malnutrition is the underlying cause of 875,000 annual child deaths, representing around 13% of deaths in children under 5 years of age globally (Black et al., 2013). Acute malnutrition is usually a result of muscle and fat loss due to illness or dietary deficits or a combination of both (Black et al., 2013). Children with acute malnutrition have compromised immunity and have a higher risk of mortality as acute malnutrition increases the risk of death from childhood illnesses such as diarrhoea (Olofin et al., 2013). Recurrent episodes of wasting can also increase the risk of linear growth faltering (Schoenbuchner et al., 2019). In addition, the risk of mortality from wasting increases when wasting and stunting occur concurrently (McDonald et al., 2013).

The high burden of acute malnutrition coupled with the slow global progress in wasting reduction warrants the implementation of evidence‐based interventions that address the problem (UNICEF, World Food Programme [WFP] et al., 2020). The community‐based management of acute malnutrition (CMAM) model is the current WHO‐recommended standard for the treatment of acute malnutrition in children aged 6–59 months (WHO, WFP et al., 2007). Although the shift to CMAM has increased coverage of the treatment of acute malnutrition, low effective coverage, frequent relapse and the high costs of the programme necessitate the implementation of both treatment and preventive interventions to accelerate the reduction of acute malnutrition. Limited evidence exists on context‐specific interventions that can be used to prevent acute malnutrition (Emergency Nutrition Network, 2018; Puett & Guerrero, 2015). Furthermore, the prevention of acute malnutrition is not given priority as the focus is on treatment (Frison et al., 2020).

Ethiopia has experienced significant progress in the reduction of malnutrition in the past decade, mainly driven by economic development and the implementation of strategies and programmes that enabled the implementation of concerted efforts to reduce malnutrition (Headey et al., 2017). Despite this progress, Ethiopia has a high burden of acute malnutrition; 10% of children under 5 years of age are wasted (Central Statistical Agency [CSA] [Ethiopia] & ICF, 2016). Additionally, in contrast to the significant declines in stunting, wasting rates have stagnated over the past two decades (CSA [Ethiopia] & ICF, 2011, 2016; CSA [Ethiopia] & ORC Macro, 2001, 2006).

Consequently, if the current trend continues, Ethiopia will not achieve the World Health Assembly (WHA) target of reducing and maintaining wasting to less than 5% by 2025 (WHO, UNICEF et al., 2018). The estimated economic burden of wasting in Ethiopia is between 150 and 225 million USD annually. The cost of supplies and human resources allocated to treat acute malnutrition are the largest contributors (40%–60%) to the economic costs associated with the treatment of acute malnutrition (Laillou et al., 2020). To achieve WHA targets, evidence‐based policies that address context‐specific risk factors of acute malnutrition are needed (UNICEF, WFP et al., 2020). However, gaps still exist in our understanding of the risk factors for wasting and interventions that can be implemented to prevent acute malnutrition in the Ethiopian context. Thus, this analysis aimed to assess the patterns of acute malnutrition and identify risk factors that have contributed to the change in acute malnutrition among children under 5 years of age between 2000 and 2016.

## 2 DATA AND METHODS

### 2.1 Data source

We use data from four rounds of the Ethiopia Demographic and Health Survey (EDHS) (2000–2016) to explore the patterns and risk factors of acute malnutrition. The Demographic and Health surveys are population‐based household health surveys that are carried out in over 90 countries globally. The availability of data for multiple rounds and standardized data collection makes these surveys suitable for exploring trends and risk factors over time. The EDHS is nationally, urban/rural and regionally representative. Children aged 0–59 months with weight measurements and information on relevant risk factors were included in the analysis.

### 2.2 Variables included in the analysis

Weight‐for‐height *z*‐score (WHZ) was the main outcome of interest in our analysis. WHZ was constructed using the 2006 WHO child growth standards. Risk factors that can potentially explain changes in WHZ were identified using the Lancet framework for action (Black et al., 2013). The framework highlights distal, intermediate, and proximal determinants of child nutritional status and nutrition‐specific and nutrition‐sensitive interventions that can address these determinants. Risk factors were included in the analysis when data were available for all four EDHS survey rounds. Proximal risk factors included in the analysis were the presence of diarrhoea, dietary diversity (including breastfeeding), and birthweight. Intermediate risk factors considered include direct and indirect nutrition intervention coverage indicators, including antenatal care (ANC) attendance, vitamin A supplementation, child vaccination and access to basic water and sanitation services.

We constructed a wealth score using pooled data from the four rounds of the EDHS and performing principal component analyses (PCAs). The variables included in the PCA were asset ownership (radio, TV, bicycle, car, electric *mitad*, lamp and electricity), cropland ownership, type of water source used, type of sanitary facility used and housing conditions. The constructed wealth index serves as a proxy measure of wealth and can be used to assess change in wealth over time. We also considered context‐specific risk factors including livelihood (pastoral) and month of data collection in the lean season (preharvest). To characterize livelihood, we constructed a cluster‐level variable using Zonal livelihood classification obtained from the CSA of Ethiopia. The CSA classifies administrative areas (zones) as majority agrarian and majority pastoralist based on livelihood. These classifications were linked to EHDS enumeration areas to explore patterns of acute malnutrition by livelihood (Agrarian vs. pastoralist). Enumeration areas are geographic areas that cover an average of 181 households. Covariates included in our models were child age, child sex, birth order, birth interval, residence, region and survey round. To account for the timing of WHZ faltering and to explore the potential contribution of age‐specific risk factors, we also performed an age disaggregated pooled regression analysis. A detailed description of variables included in the analysis is provided in Supporting Information: Table 1S.

### 2.3 Statistical analysis

Data management and statistical analysis were conducted in Stata Version 16.0. Sampling weights were applied when estimating prevalence to account for the cluster sampling used in the EDHS. We initially performed a regression analysis using pooled data from all four EDHS survey rounds to identify the nature and magnitude of the relationship between risk factors and WHZ. Then to assess the contributions of risk factors to the change in WHZ between 2000 and 2016, we performed a Blinder–Oaxaca decomposition (Jann, 2008). This method decomposes the change in WHZ between 2000 and 2016 into two parts: a part that is due to the difference in the distribution of risk factors (covariates effect), and the second part that is due to the difference in the effect of these risk factors (coefficients effect).

The mean outcome difference to be explained ($∆\overset{®}{Y}$) is then simply the difference between the mean outcomes for observations in 2000 (${\overset{®}{Y}}_{2000}$) and 2016 (${\overset{®}{Y}}_{2016}$):

$$∆\overset{®}{Y}=({\overset{®}{Y}}_{2016}-{\overset{®}{Y}}_{2000}).$$

The mean outcome difference in the two portions is expressed as

$$∆\bar{Y}=({\bar{X}}_{2016}-{\bar{X}}_{2000})\prime \beta^{*}+[{\bar{X}}_{2016}^{\prime }\left(\beta_{2016}-\beta^{*}\right)+{\bar{X}}_{2000}^{\prime }(\beta^{*}-\beta_{2000})].$$

The first portion of the difference in the means is attributable to the explanatory variables/endowments, which is also known as the explained portion and is weighted by the vector of pooled regression coefficients ($\beta^{*}$). The second term is the coefficient effect, which is also known as the unexplained or discriminatory portion.

## 3 RESULTS

A total of 21,923 children aged 0–59 months drawn from the four rounds of the EDHS were included in the analysis. Our sample was a subset of the EDHS and included the youngest, live child for whom anthropometric data were available. Table 1 presents the trends in acute malnutrition and risk factors between 2000 and 2016 for these children. Mean WHZ increased by 0.28 standard deviations between 2000 and 2016. Wasting declined by 4.4%. The prevalence of diarrhoea, a risk factor for acute malnutrition, significantly declined (14% reduction) between 2000 and 2016. Perceived low weight at birth declined by 9% in the same period. The proportion of children whose mothers received four or more ANC visits during pregnancy roughly doubled. Child vitamin A supplementation declined by 12%. Access to basic water and sanitation services improved (although the increase in basic sanitation coverage was modest at 5%). Both mean wealth score (92% change) and secondary or higher educational attainment increased between 2000 and 2016.

**Table 1:** Trends in wasting and potential risk factors among children aged 0–59 months

Variables	2000 (*n* = 6251)	2005 (*n* = 2696)	2011 (*n* = 6729)	2016 (*n* = 6247)	Change (2016–2000)	*p* Value for the change
WHZ, mean (SD)	−0.73 (1.4)	−0.47 (1.7)	−0.57 (1.2)	−0.45 (1.3)	0.28 (1.4)	<0.001
Wasting (%)	15.6	15.0	11.7	11.2	−4.4	<0.001
MAM (%)	10.0	8.5	8.0	7.8	−2.2	0.002
SAM (%)	5.6	6.5	3.6	3.4	−2.2	<0.001
Concurrent wasting and stunting (%)	8.4	6.4	4.5	3.1	−5.3	<0.001
Recent diarrhoea (%)	29.0	22.7	16.8	14.6	−14.4	<0.001
Perceived low weight at birth (%)	36.3	29.6	31.6	26.9	−9.4	<0.001
Child received vitamin A supplements	55.5	49.6	50.4	43.2	−12.3	0.135
Attended 4+ ANC visits (%)	10.6	12.8	19.1	32.0	21.4	<0.001
Basic water (%)	11.6	44.6	30.5	43.4	31.8	<0.001
Basic sanitation (%)	0.1	4.4	7.9	5.6	5.5	<0.001
Livelihood: Pastoral (%)	1.7	1.2	2.6	3.8	2.1	<0.001
Wealth score (mean)	1.3 (1.4)	1.5 (1.5)	2.2 (1.7)	2.5 (1.9)	1.2	<0.001
Month of interview: Lean season (%)	0.4	61.0	—	0.8	0.4	0.022
Maternal education: Second/higher (%)	5.5	5.3	4.4	8.4	2.9	<0.001

Abbreviations: ANC, antenatal care; MAM, moderate acute malnutrition; SAM, Severe acute malnutrtion; WHZ, weight‐for‐height *z*‐score.

**Figure 1:**
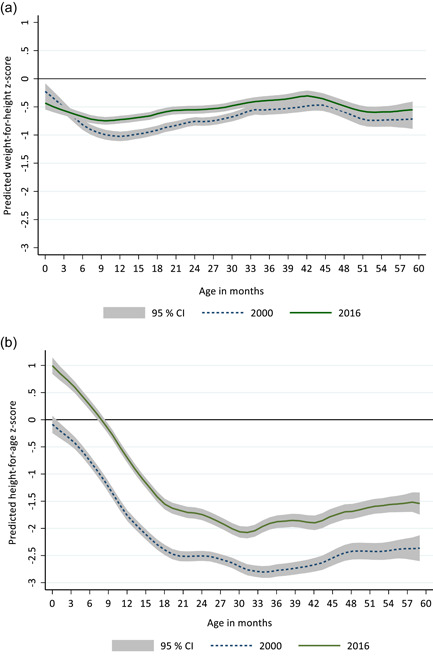
(a) Mean weight‐for‐height *z*‐scores by child age (months). (b) Mean height‐for‐age *z*‐scores by child age (months). The horizontal line at 0 represents the median of the World Health Organization (WHO) child growth standards. CI, confidence interval.

Figure 1a shows patterns in mean WHZ by age (birth to 59 months) in 2000 and 2016 relative to the WHO child growth standards. The relationship between age and WHZ followed a similar pattern in both 2000 and 2016. The sharpest decline by age in WHZ was from birth to 6 months. Mean WHZ was lowest around 9–12 months. Although mean WHZ at birth was lower in 2016 compared with 2000, the decrease in mean WAZ from birth to 6 months was less marked in 2016. The mean WHZ for Ethiopian children was below the reference (WHO) from birth to 59 months in both 2000 and 2016. In contrast, the mean of WHZ, height‐for‐age *z*‐score (HAZ) was above the reference at birth (2016), with a significant decrease in mean HAZ occurring after 6 months of age (Figure 1b). Additionally, the increase in mean WHZ between 2000 and 2016 was lower compared with the increase in mean HAZ during the same period. Equity analysis showed that although differences in wasting prevalence between the poorest and wealthiest households have declined over time, significant economic inequities in wasting still exist. However, urban–rural inequities were less prevalent with the absolute difference in wasting between children who reside in urban and rural settings decreasing between 2000 and 2016 (Supporting Information: Figure 2S and Table 2S).

We identified significant predictors of WHZ using pooled linear regression models (2000–2016). We included 20,651 children who had complete data for all predictors in this analysis. Table 2 presents age disaggregated regression results for children aged 0–5, 6–23 and 24–59 months. Among children aged 0–5 months, perceived low weight at birth, recent diarrhoea, exclusive breastfeeding, and access to basic water were significant predictors of WHZ. The presence of diarrhoea at any time during the 2 weeks preceding the survey was associated with a 0.255 decline in WHZ (*p* = 0.005). Perceived low weight at birth was associated with a 0.154 decline in WHZ (*p* = 0.02). Exclusive breastfeeding for the first 6 months was associated with a 0.174 increase in WHZ (*p* = 0.012). Access to basic water also predicted an increase in WHZ (*β* [95% CI]: 0.185 [0.031,0.339], *p* = 0.018). Perceived low weight at birth (β [95% CI]: −0.31, [−0.373,−0.248], *p* < 0.001) and diarrhoea (β [95% CI]: −0.244 [−0.309,−0.179], *p* < 0.001) continued to be significant predictors of WHZ among children aged 6–23 months. Access to basic sanitation facilities, receiving at least four ANC visits, wealth, month of data collection and maternal education were additional risk factors that were associated with WHZ in this age group. Access to basic sanitation was associated with a 0.177 increase in WHZ (*p *= 0.005) while receiving four or more ANC visits predicted a 0.081 increase in WHZ (*p* = 0.033). A unit increase in wealth score predicted a 0.111 increase in WHZ (*p* < 0.001). An interview month that falls in the lean or preharvest season predicted a 0.233 increase in WHZ (*p* = 0.006). Age‐specific risk factors included in the 6–23‐month model, namely, age‐appropriate vaccination and safe disposal of child stool, did not significantly predict WHZ. To explore if dietary diversity is a predictor of WHZ, we conducted an additional analysis for children aged 6–23 months pooling the EDHS rounds that have data on dietary diversity (2005–2016) (Supporting Information: Table 4S). Our findings show that dietary diversity was not a significant predictor of WHZ. Similar to our findings in children aged 6–23 months, among children aged 24–59 months, perceived low weight at birth, recent diarrhoea, receiving four or more ANC visits, wealth and maternal education were significant predictors of WHZ. However, access to basic sanitation was a significant predictor of WHZ in children aged 6–23 months but not in 24–59 months. Additional information on pooled regression results is included in the Supporting Information: Table 3S–7S.

**Table 2:** Pooled linear regression analysis to identify risk factors of WHZ in children, 2000–2016^a^

	0–5 months (*n* = 2879)			6–23 months (*n* = 8638)			24–59 months (*n* = 9129)		
	*β*	95% CI	*p* Value	*β*	95% CI	*p* Value	*β*	95% CI	*p* Value
Perceived low weight at birth	−0.154	[−0.285,−0.024]	0.02	−0.31	[−0.373,−0.248]	<0.001	−0.281	[−0.336,−0.226]	<0.001
Recent diarrhoea	−0.255	[−0.434,−0.077]	0.005	−0.244	[−0.309,−0.179]	<0.001	−0.189	[−0.259,−0.119]	<0.001
Child is exclusively breastfed	0.174	[0.038,0.311]	0.012						
Child received vitamin A supplements				0.013	[−0.048,0.075]	0.672	0.044	[−0.007,0.094]	0.093
Basic water	0.185	[0.031,0.339]	0.018	−0.052	[−0.124,0.020]	0.159	0.016	[−0.046,0.077]	0.616
Basic sanitation	0.216	[−0.069,0.501]	0.137	0.177	[0.055,0.299]	0.005	0.04	[−0.055,0.136]	0.407
Attended 4+ ANC visits	0.072	[−0.102,0.246]	0.416	0.081	[0.007,0.155]	0.033	0.078	[0.018,0.138]	0.011
Wealth score (0–10)	−0.001	[−0.069,0.067]	0.984	0.111	[0.080,0.142]	<0.001	0.055	[0.031,0.080]	<0.001
Livelihood: Pastoral	−0.057	[−0.417,0.302]	0.754	0.141	[‐0.031,0.312]	0.107	−0.084	[−0.222,0.055]	0.237
Month of interview: Lean season	0.218	[−0.186,0.621]	0.29	0.233	[0.067,0.400]	0.006	0.156	[0.006,0.305]	0.041
Maternal education: Secondary/higher	0.131	[−0.117,0.379]	0.299	0.139	[0.023,0.256]	0.019	0.153	[0.054,0.251]	0.002
Age‐appropriate vaccination				0.04	[−0.033,0.114]	0.284			
Safe disposal of child stools				0.062	[−0.008,0.132]	0.08			

Abbreviations: ANC, antenatal care; CI, confidence interval; WHZ, weight‐for‐height *z*‐score.

^a^ Beta coefficients (95% CI) are estimated using linear regression with a robust variance estimator. Models were adjusted for child age, child sex, birth order, residence, region and survey round.

**Figure 2:**
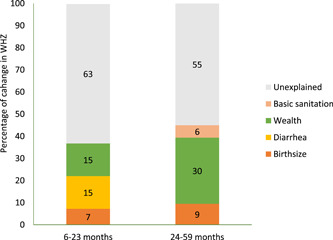
Estimated contribution of risk factors to change in weight‐for‐height *z*‐score between 2000 and 2016

Figure 2 presents the results of Oaxaca decomposition analysis performed to estimate the contribution of changes in these risk factors to the change in WHZ between 2000 and 2016. As WHZ did not significantly change between 2000 and 2016 among children aged 0–5 months, we did not perform a change decomposition analysis in this age group. In children aged 6–23 months, an improvement in socioeconomic status (wealth score) accounted for 15% of the increase in WHZ. The reduction in the prevalence of diarrhoea between 2000 and 2016 also accounted for 15% of the change in WHZ. The modest decline in perceived low weight at birth accounted for some of the changes in WHZ (7%). We found similar results for children aged 24–59 months, with the exception that access to basic sanitation accounted for 6% of the change in WHZ in children aged 24–59 months. Additionally, decline in diarrhoea did not explain the change in WHZ in older children (24–59 months). The factors included in our model accounted for 37%–45% of the change in WHZ. The proportion of change in WHZ accounted for by the risk factors included in the analysis varied with age with more change accounted for in older children (24–59 months).

## 4 DISCUSSION

This study utilized data from multiple rounds of a large‐scale nationally representative survey to describe patterns and risk factors of acute malnutrition. Our findings show a modest increase in mean WHZ between 2000 and 2016. Mean WHZ sharply decreased from birth to 6 months of age. Perceived low weight at birth and recent diarrhoea predicted a decrease in WHZ among children aged 0–5, 6–23 and 23–59 months. In contrast, WHZ increased with an increase in wealth, maternal education, ANC visits and access to basic sanitation. However, only about half (37%–45%) of the improvement in WHZ between 2000 and 2016 was accounted for by the change in the risk factors included in our analysis.

Globally limited progress has been made towards the WHA target of keeping wasting prevalence below 5%. Only about a quarter of countries are on track to reach this target (UNICEF, WHO et al., 2020). In Ethiopia, the prevalence of wasting has stagnated at around 10% (CSA [Ethiopia] & ICF, 2016), and it continues to be a public health problem. Additionally, this cross‐sectional prevalence of wasting likely underestimates the burden of wasting as it does not capture incidence (Bulti et al., 2017; Deconinck et al., 2016). Although wasting is commonly thought to be a transient condition that can be reversed with treatment, growing evidence suggests that it might have long‐term consequences. Recent studies that explore the relationship between wasting and stunting have found that wasted children are at a higher risk of subsequent stunting (Dewey et al., 2005; Richard et al., 2012; Schoenbuchner et al., 2019). A review of growth monitoring records of Gambian children over four decades by Schoenbuchner et al. (2019) found that wasted children were more vulnerable to repeated episodes of wasting and progression to stunting. Thus, the lack of progress in wasting reduction can also affect progress in the reduction of stunting.

Understanding the relationship between WHZ and age can aid the identification of critical windows of opportunity for the implementation of interventions to prevent wasting (Bhutta et al., 2017). We found that a sharp decrease in mean WHZ occurs in the first 6 months of life in children under 5 years of age. This pattern is similar to what has been reported globally (Victora et al., 2021). Additionally, recent evidence generated from longitudinal studies found that the incidence of wasting was highest from birth to 3 months (Mertens et al., 2020). These findings highlight the importance of nutrition interventions during pregnancy and early life for the prevention of acute malnutrition.

The aetiology of wasting is rather complex as multiple context‐specific risk factors are at play, and these factors are poorly understood (Bhutta et al., 2017). Existing evidence shows that wasting at birth and early childhood is strongly associated with low birthweight (Christian et al., 2013). Maternal nutrition during fetal growth is likely an important risk factor for low birthweight as characteristics such as maternal education, and low body mass index have been associated with wasting at birth (Victora, 2015). In our analysis, low birth size was a significant predictor of WHZ across age groups; emphasizing the importance of optimal nutrition during pregnancy and post‐natal interventions to manage low birthweight. Recent diarrhoeal disease and access to basic sanitation services were other significant risk factors that predicted WHZ. Access to poor water, sanitation and hygiene (WASH) services have been shown to increase the risk of infectious disease such as diarrhoea, resulting in wasting. Additionally, poor sanitary conditions and resulting infections can also contribute to the development of environmental enteric dysfunction, which will lead to reduced nutrient absorption, reduced appetite and food intake (Colombara et al., 2016). Environmental factors such as drought and seasonal changes in food security and infection can lead to a decrease in WHZ. Food supply is low during the lean/preharvest season, suggesting that WHZ will be lower at this time. Surprisingly we found that the collection of data during the lean months predicted an increase in WHZ. However, similar findings have been reported for Ethiopia, suggesting that the effects of lower food intake in the preharvest season will likely be reflected in WHZ during the harvest season (Roba et al., 2016). Vitamin A supplementation and dietary diversity did not significantly predict WHZ nor drive change in WHZ in our analysis. This may be due to the lack of sustained improvements in these indicators in Ethiopia rather than a lack of relationship with WHZ.

Wealth was the main driver of the change in WHZ in our analysis. Thus, along with direct and indirect nutrition interventions, narrowing economic inequities can lead to better prevention and management of acute malnutrition. Although urban residence was associated with an increase in WHZ across all age groups, we found that the absolute difference in wasting prevalence between children who reside in urban and rural settings decreased between 2000 and 2016. The implementation of the CMAM and subsequent increase in the coverage of the treatment of severe acute malnutrition might have contributed to the decline in prorural inequities.

Age disaggregated analysis of risk factors for WHZ showed that the magnitude of association between wealth, access to basic sanitation services and ANC visits (a proxy indicator for access to health services) were stronger for younger children (6–23 months) compared with older children (24–59 months). Similar findings have been reported by Alderman and Headey (2018) who used demographic and health survey data from 125 countries and found that the magnitude of association of wealth and improved toilets with WHZ was larger for children aged 0–23 months compared with children aged 24–59 months. Thus, our findings emphasize the importance of intervening in the first 1000 days to address underlying risk factors of acute malnutrition. Basic water services use was significantly associated with WHZ only among children aged 0–5 months. This finding is surprising as we would also expect an association between access to safe water and WHZ for children aged 6–23 years as complementary feeding typically starts after 6 months of age and the prevalence of diarrhoea is high at this age for Ethiopian children (Girma et al., 2021).

This study has several limitations. First, our analysis is based on risk factors available in the EDHS, which are not comprehensive. Information on important risk factors such as drought, seasonal changes in food security, infection, food intake and utilization of acute malnutrition treatment services is not collected in population‐based surveys such as the EDHS. A larger percentage of change in WHZ would likely have been accounted for if more risk factors were included in this analysis. Second, although the EDHS surveys are ideal for tracking long‐term trends in WHZ, the cross‐sectional design of these surveys does not capture the dynamic nature of acute malnutrition. Multiple risk factors can be involved in the pathway to acute malnutrition in a given context, and the combination of these factors is likely to change over time (Akombi et al., 2017; Bhutta et al., 2017)). These changes might not have been captured in our analysis. Furthermore, acute malnutrition can vary over time and within an individual. Longitudinal studies have shown that recurrent episodes of wasting can happen in the same child in a given period. Thus, it is likely that the cross‐sectional measurement does not fully depict the burden of the wasting.

Despite these limitations, we present evidence on the patterns and risk factors of acute malnutrition using robust nationally representative data. Our findings also highlight data gaps to identify context‐specific risk factors and thus intervention to prevent wasting. We also show that pregnancy and the first 6 months of birth are critical time points to prevent wasting. This finding reemphasizes the importance of improving maternal nutrition to reduce low birthweight and prevent wasting. Specific interventions that can be implemented at this stage include increased coverage of iron/folic acid supplementation during pregnancy and targeted supplemental feeding for vulnerable pregnant women. Additionally, coverage of basic WASH services should be scaled up along with coverage of oral rehydration solution and zinc treatment to manage diarrhoeal diseases.

A key finding of our analysis was that more than 50% of the improvement in WHZ was not accounted for by the change in commonly measured risk factors. A recent review of the state of evidence on wasting prevention highlighted the need for more longitudinal studies that measure individual‐level nutritional status overtime to elucidate causal pathways and identify preventive interventions (Frison et al., 2020). Such data would enable us to take into account the relationship between risk factors over time and capture the seasonal patterns in exposure to risk factors. More evidence can also be generated by utilizing routine monitoring data, collecting information on additional risk factors in cross‐sectional studies and implementing well‐designed programme evaluations.

## AUTHOR CONTRIBUTIONS

Meron Girma, Arnaud Laillou and Kaleab Baye performed the research with help from Alemayehu Hussein, Aregash Samuel, Cornelia van Zyl, Masresha Tessema and Stanley Chitekwe. Alemayehu Hussein analysed the data with help from Meron Girma. All authors took responsibility for reviewing, final editing and approval of the manuscript.

## CONFLICT OF INTEREST

The authors declare no conflict of interest.

## Supporting information

Supporting information.

## Data Availability

All our analyses are based on Demographic and Health Surveys, which are available at the Measure DHS website after appropriate registration: http://dhsprogram.com/data/available-datasets.cfm.

## References

[mcn13392-bib-0001] Akombi, B. J. , Agho, K. E. , Hall, J. J. , Wali, N. , Renzaho, A. M. N. , & Merom, D. (2017). Stunting, wasting and underweight in sub‐Saharan Africa: A systematic review. International Journal of Environmental Research and Public Health, 14(8), 863. 10.3390/ijerph14080863 28788108 PMC5580567

[mcn13392-bib-0002] Alderman, H. , & Headey, D. (2018). The timing of growth faltering has important implications for observational analyses of the underlying determinants of nutrition outcomes. PLoS One, 13(4), e0195904. 10.1371/journal.pone.0195904 29694431 PMC5919068

[mcn13392-bib-0003] Bhutta, Z. A. , Berkley, J. A. , Bandsma, R. H. J. , Kerac, M. , Trehan, I. , & Briend, A. (2017). Severe childhood malnutrition. Nature Reviews Disease Primers, 3, 17067. 10.1038/nrdp.2017.67 PMC7004825 28933421

[mcn13392-bib-0004] Black, R. E. , Victora, C. G. , Walker, S. P. , Bhutta, Z. A. , Christian, P. , de Onis, M. , & Uauy, R. (2013). Maternal and child undernutrition and overweight in low‐income and middle‐income countries. The Lancet, 382(9890), 427–451. 10.1016/s0140-6736(13)60937-x 23746772

[mcn13392-bib-0005] Bulti, A. , Briend, A. , Dale, N. M. , De Wagt, A. , Chiwile, F. , Chitekwe, S. , Isokpunwu, C. , & Myatt, M. (2017). Improving estimates of the burden of severe acute malnutrition and predictions of caseload for programs treating severe acute malnutrition: Experiences from Nigeria. Archives of Public Health, 75, 66. 10.1186/s13690-017-0234-4 29152260 PMC5679511

[mcn13392-bib-0006] Central Statistical Agency [Ethiopia] and ICF . (2011). Ethiopia demographic and health survey 2011.

[mcn13392-bib-0007] Central Statistical Agency [Ethiopia] and ICF . (2016). Ethiopia demographic and health survey 2016.

[mcn13392-bib-0008] Central Statistical Agency [Ethiopia] and ORC Macro . (2001). Ethiopia demographic and health survey 2000.

[mcn13392-bib-0009] Central Statistical Agency [Ethiopia] and ORC Macro . (2006). Ethiopia demographic and health survey 2005.

[mcn13392-bib-0010] Christian, P. , Lee, S. E. , Donahue Angel, M. , Adair, L. S. , Arifeen, S. E. , Ashorn, P. , Barros, F. C. , Fall, C. H. , Fawzi, W. W. , Hao, W. , Hu, G. , Humphrey, J. H. , Huybregts, L. , Joglekar, C. V. , Kariuki, S. K. , Kolsteren, P. , Krishnaveni, G. V. , Liu, E. , Martorell, R. , … Black, R. E. (2013). Risk of childhood undernutrition related to small‐for‐gestational age and preterm birth in low‐ and middle‐income countries. International Journal of Epidemiology, 42(5), 1340–1355. 10.1093/ije/dyt109 23920141 PMC3816349

[mcn13392-bib-0011] Colombara, D. V. , Khalil, I. A.‐M. , Rao, P. C. , Troeger, C. , Forouzanfar, M. H. , Riddle, M. S. , & Mokdad, A. H. (2016). Chronic health consequences of acute enteric infections in the developing world. The American Journal of Gastroenterology Supplements, 3(2), 4–11. 10.1038/ajgsup.2016.9

[mcn13392-bib-0012] Deconinck, H. , Pesonen, A. , Hallarou, M. , Gerard, J. C. , Briend, A. , Donnen, P. , & Macq, J. (2016). Challenges of estimating the annual caseload of severe acute malnutrition: The case of Niger. PLoS One, 11(9), e0162534. 10.1371/journal.pone.0162534 27606677 PMC5015826

[mcn13392-bib-0013] Dewey, K. G. , Hawck, M. G. , Brown, K. H. , Lartey, A. , Cohen, R. J. , & Peerson, J. M. (2005). Infant weight‐for‐length is positively associated with subsequent linear growth across four different populations. Maternal & Child Nutrition, 1(1), 11–20. 10.1111/j.1740-8709.2004.00004.x 16881875 PMC6874388

[mcn13392-bib-0014] Emergency Nutrition Network (ENN) . (2018). The current state of evidence and thinking on wasting prevention.

[mcn13392-bib-0015] Frison, S. , Angood, C. , Khara, T. , Bahwere, P. , Black, R. E. , & Briend, A. , Wasting Prevention Working Group Collaborators . (2020). Prevention of child wasting: Results of a Child Health & Nutrition Research Initiative (CHNRI) prioritisation exercise. PLoS One, 15(2), e0228151. 10.1371/journal.pone.0228151 32049994 PMC7015423

[mcn13392-bib-0016] Girma, M. , Hussein, A. , Norris, T. , Genye, T. , Tessema, M. , Bossuyt, A. , Hadis, M. , Zyl, C. , Goyol, K. , & Samuel, A. (2021). Progress in water, sanitation and hygiene (WASH) coverage and potential contribution to the decline in diarrhea and stunting in Ethiopia. *Maternal & Child Nutrition*, e13280. 10.1111/mcn.13280 PMC11258769 34738323

[mcn13392-bib-0017] Headey, D. , Hoddinott, J. , & Park, S. (2017). Accounting for nutritional changes in six success stories: A regression‐decomposition approach. Global Food Security, 13, 12–20. 10.1016/j.gfs.2017.02.003

[mcn13392-bib-0018] Jann, B. (2008). The Blinder–Oaxaca decomposition for linear regression models. The Stata Journal: Promoting Communications on Statistics and Stata, 8(4), 453–479. 10.1177/1536867x0800800401

[mcn13392-bib-0019] Laillou, A. , Baye, K. , Meseret, Z. , Darsene, H. , Rashid, A. , & Chitekwe, S. (2020). Wasted children and wasted time: A challenge to meeting the nutrition sustainable development goals with a high economic impact to Ethiopia. Nutrients, 12(12), 3698. 10.3390/nu12123698 33266008 PMC7760409

[mcn13392-bib-0020] McDonald, C. M. , Olofin, I. , Flaxman, S. , Fawzi, W. W. , Spiegelman, D. , Caulfield, L. E. , Ezzati, M. , & Danaei, G. , Nutrition Impact Model Study . (2013). The effect of multiple anthropometric deficits on child mortality: Meta‐analysis of individual data in 10 prospective studies from developing countries. American Journal of Clinical Nutrition, 97(4), 896–901. 10.3945/ajcn.112.047639 23426036

[mcn13392-bib-0021] Mertens, A. , Benjamin‐Chung, J. , Colford, J. M. , Hubbard, A. E. , van der Laan, M. J. , Coyle, J. , & Arnold, B. F. (2020). Child wasting and concurrent stunting in low‐ and middle‐income countries. *medRXiv*. 10.1101/2020.06.09.20126979 PMC10511327 37704720

[mcn13392-bib-0022] Olofin, I. , McDonald, C. M. , Ezzati, M. , Flaxman, S. , Black, R. E. , Fawzi, W. W. , & Danaei, G. , Nutrition Impact Model, S . (2013). Associations of suboptimal growth with all‐cause and cause‐specific mortality in children under five years: A pooled analysis of ten prospective studies. PLoS One, 8(5), e64636. 10.1371/journal.pone.0064636 23734210 PMC3667136

[mcn13392-bib-0023] Puett, C. , & Guerrero, S. (2015). Barriers to access for severe acute malnutrition treatment services in Pakistan and Ethiopia: A comparative qualitative analysis. Public Health Nutrition, 18(10), 1873–1882. 10.1017/S1368980014002444 26017477 PMC10271649

[mcn13392-bib-0024] Richard, S. A. , Black, R. E. , Gilman, R. H. , Guerrant, R. L. , Kang, G. , Lanata, C. F. , Mølbak, K. , Rasmussen, Z. A. , Sack, R. B. , Valentiner‐Branth, P. , Checkley, W. , & Childhood Infection and Malnutrition Network . (2012). Wasting is associated with stunting in early childhood. Journal of Nutrition, 142(7), 1291–1296. 10.3945/jn.111.154922 22623393 PMC3374667

[mcn13392-bib-0025] Roba, K. T. , O'Connor, T. P. , Belachew, T. , & O'Brien, N. M. (2016). Variations between post‐ and pre‐harvest seasons in stunting, wasting, and Infant and Young Child Feeding (IYCF) practices among children 6‐23 months of age in lowland and midland agro‐ecological zones of rural Ethiopia. The Pan African Medical Journal, 24, 163. 10.11604/pamj.2016.24.163.9387 27795761 PMC5072826

[mcn13392-bib-0026] Schoenbuchner, S. M. , Dolan, C. , Mwangome, M. , Hall, A. , Richard, S. A. , Wells, J. C. , Khara, T. , Sonko, B. , Prentice, A. M. , & Moore, S. E. (2019). The relationship between wasting and stunting: A retrospective cohort analysis of longitudinal data in Gambian children from 1976 to 2016. American Journal of Clinical Nutrition, 110(2), 498–507. 10.1093/ajcn/nqy326 30753251 PMC6669055

[mcn13392-bib-0027] United Nations Children's Fund, World Food Programme, WHO, United Nations High Commissioner for Refugees, & Food and Agriculture Organization of the United Nations (UNICEF, WFP, WHO, UNHCR, & FAO) . (2020). *Global Action Plan (GAP) on child wasting: A framework for action*.

[mcn13392-bib-0028] United Nations Children's Fund, World Health Organization, & International Bank for Reconstruction and Development/The World Bank (UNICEF, WHO, & World Bank) . (2020). Levels and trends in child malnutrition: Key findings of the 2020 Edition of the Joint Child Malnutrition Estimates.

[mcn13392-bib-0029] Victora, C. G. , Christian, P. , Vidaletti, L. P. , Gatica‐Domínguez, G. , Menon, P. , & Black, R. E. (2021). Revisiting maternal and child undernutrition in low‐income and middle‐income countries: Variable progress towards an unfinished agenda. The Lancet, 397(10282), P1388–P1399. 10.1016/s0140-6736(21)00394-9 PMC7613170 33691094

[mcn13392-bib-0040] Victora, C. G. , Villar, J. , Barros, F. C. , Ismail, L. C. , Chumlea, C. , Papageorghiou, A. T. , Bertino, E. , Ohuma, E. O. , Lambert, A. , Carvalho, M. , Jaffer, Y. A. , Altman, D. G. , Noble, J. A. , Gravett, M. G. , Purwar, M. , Frederick, I. O. , Pang, R. , Bhutta, Z. A. , & Kennedy, S. H . (2015). Anthropometric characterization of impaired fetal growth. JAMA Pediatrics, 169(7), e151431. 10.1001/jamapediatrics.2015.1431 26147058

[mcn13392-bib-0030] World Health Organization, United Nations Children's Fund, World Food Programme, & European Union (WHO, UNICEF, & EU) . (2018). *Global targets 2025: Ethiopia country progress report*.

[mcn13392-bib-0031] World Health Organization, World Food Programme, United Nations System Standing Committee on Nutrition, & United Nations Children's Fund (WHO, WFP, & UNICEF) . (2007). *Community‐based management of severe acute malnutrition*.

