# Supplementary material for: Drivers of change in weight‐for‐height among children under 5 years of age in Ethiopia: Risk factors and data gaps to identify risk factors
Source: Matern Child Nutr. 2022 Jun 20;20(Suppl 5):e13392. doi: 10.1111/mcn.13392 (PMC11258770; doi:10.1111/mcn.13392)
Supplement: Supplementary file 1 — Supporting information. [file MCN-20-e13392-s001.docx]

**Table 1S. Description of Variables Used in the decomposition Analysis**

| **Variables** | **Definition** |
| --- | --- |
| Recent Diarrhea | Percentage of living children (0-59 months) with diarrhea (three or more loose stools per day) at any time in the two weeks preceding the survey. |
| Perceived low weight at birth | Percentage of live births to interviewed women in the 5 years preceding the survey where the mother’s estimated baby’s size at time of birth as smaller than average. |
| MDD-5 | Percentage of children aged 6-23 months fed a minimum dietary diversity of 5 out of 8 food groups during the day or night preceding the survey. |
| Vitamin A supplementation | Percentage children who received two doses of vitamin A supplements in the past 6 months |
| Age appropriate vaccination | Percentage of children aged 6-23 months who have received BCG, 3 doses of DPT, 3 doses of polio, and 1 dose of MCV vaccines. |
| Four or more ANC visits | Percentage of women who had 4 or more ANC visits for the most recent birth. |
| Basic water | Percentage of households whose main source of drinking water is from an improved source, provided collection time is not more than 30 minutes for a round trip, including queuing. |
| Basic sanitation | Percentage of households who use improved toilet facilities that are not shared with other households. |
| Safe stool disposal | Percentage of children whose stools are disposed of appropriately. |
| Wealth score | A wealth score constructed for the pooled data (2000,2005, 2011 and 2016). |
| Maternal education: Secondary or higher | Percentage of women who have attained secondary or higher education |
| Livelihood | An enumeration area level clustering variable that classifies EA’s as majority agrarian or pastoralist livelihood zones. |
| Month of data collection: Lean season | Data collection took place during the months before the main agricultural harvest season also referred to as lean season. The lean season falls during May-September |
| Month of birth | Mother was in third trimester of pregnancy during lean-season (May-September) for the index child |
| Child age | Child age in months. |
| Child sex | Child sex. |
| Birth order | The order number of the births from first to last. |
| Residence | Urban vs rural |
| Region | Region of residence |
| Survey round | Year of EDHS survey |

**Figure 1S.** Change over time in wasting by a) Wealth. Dots show prevalence of wasting across wealth quintiles (poorest, poorer, middle, wealthier and wealthiest). b) Residence. Dots show the prevalence of wasting among rural (green) and urban (red) residents

a) Wealth b) Residence

**Table 2S**. Slope index of inequality (SII) estimated for wasting by wealth and residence.

|  | Wealth quantile | | | |  | | Residence | | | |
| --- | --- | --- | --- | --- | --- | --- | --- | --- | --- | --- |
|  | SII | 95% CI | p-value |  | | SII | | 95% CI | p-value |  |
| 2000 | -2 | [-6, 2] | 0.362 |  | | 0.11 | | [3,2] | 0.009 |  |
| 2005 | -11 | [-17,-5] | <0.001 |  | | 0.11 | | [-1,2] | 0.066 |  |
| 2011 | -12 | [-16,-7] | <0.001 |  | | 0.14 | | [4,2] | 0.007 |  |
| 2016 | -9 | [-14,-4] | <0.001 |  | | 0.01 | | [-5,8] | 0.699 |  |

SII: quantifies absolute difference in wasting prevalence between the wealthiest and the poorest and between urban and rural residence. Greater SII values represent higher levels of inequality. The SII is equal to zero if there is no inequality.

**Table 3S**. Full Pooled linear regression model for risk factors of WHZ in children age 0-5 months (n= 2879), 2000-2016

|  | 0-5 months  n=2,879 | | |
| --- | --- | --- | --- |
|  | B | 95% CI | P-value |
| Perceived low weight at birth | -0.154 | [-0.285, -0.024] | 0.02 |
| Recent diarrhea | -0.255 | [-0.434, -0.077] | 0.005 |
| Child is exclusively breastfed | 0.174 | [0.038, 0.311] | 0.012 |
| Basic Water | 0.185 | [0.031, 0.339] | 0.018 |
| Basic sanitation | 0.216 | [-0.069, 0.501] | 0.137 |
| Attended 4+ ANC visits | 0.072 | [-0.102, 0.246] | 0.416 |
| Wealth score (0-10) | -0.001 | [-0.069, 0.067] | 0.984 |
| Livelihood: Pastoral | -0.057 | [-0.417, 0.302] | 0.754 |
| Month of interview: Lean season | 0.218 | [-0.186, 0.621] | 0.29 |
| Maternal education: Secondary/higher | 0.131 | [-0.117, 0.379] | 0.299 |
| Child sex | 0.052 | [-0.069, 0.174] | 0.4 |
| Child age | -0.051 | [-0.115,0.135] | 0.122 |
| Birth order | -0.002 | [-0.027, 0.024] | 0.9 |
| Residence | 0.275 | [-0.055, 0.605] | 0.102 |
| Region: Tigray (Reference) |  |  |  |
| Afar | -0.265 | [-0.639, 0.110] | 0.166 |
| Amhara | 0.208 | [-0.064, 0.480] | 0.133 |
| Oromia | 0.345 | [0.110, 0.580] | 0.004 |
| Somali | -0.039 | [-0.382, 0.303] | 0.822 |
| Benishangul | 0.001 | [-0.287, 0.289] | 0.995 |
| SNNPR | 0.241 | [0.008, 0.474] | 0.043 |
| Gambela | -0.149 | [-0.461, 0.163] | 0.348 |
| Harari | -0.108 | [-0.424, 0.208] | 0.503 |
| Addis Ababa | 0.052 | [-0.344, 0.449] | 0.796 |
| Dire Dawa | -0.423 | [-0.719, -0.127] | 0.005 |
| Year : 2000 (Reference) |  |  |  |
| 2005 | -0.323 | [-0.703, 0.056] | 0.095 |
| 2011 | -0.184 | [-0.359, -0.009] | 0.04 |
| 2016 | -0.141 | [-0.332, 0.050] | 0.147 |

**Table 4S**. Full Pooled linear regression model for risk factors of WHZ in children age 6-23 months (n= 8638), 2000-2016

|  |  | 6-23 months |  |
| --- | --- | --- | --- |
|  |  | n=8,638 |  |
|  | B | 95% CI | P-value |
| Perceived low weight at birth | -0.310 | [-0.373, -0.248] | <0.001 |
| Recent diarrhea | -0.244 | [-0.309, -0.179] | <0.001 |
| Child received vitamin A supplements | 0.013 | [-0.048, 0.075] | 0.672 |
| Basic water | -0.052 | [-0.124, 0.020] | 0.159 |
| Basic Water | 0.177 | [0.055, 0.299] | 0.005 |
| Attended 4+ ANC visits | 0.081 | [0.007, 0.155] | 0.033 |
| Wealth score (0-10) | 0.111 | [0.080, 0.142] | <0.001 |
| Livelihood: Pastoral | 0.141 | [-0.031, 0.312] | 0.107 |
| Month of interview: Lean season | 0.233 | [0.067, 0.400] | 0.006 |
| Maternal education: Secondary/higher | 0.139 | [0.023, 0.256] | 0.019 |
| Age appropriate vaccination | 0.040 | [-0.033, 0.114] | 0.284 |
| Safe disposal of child stools | 0.062 | [-0.008, 0.132] | 0.08 |
| Child sex | 0.188 | [0.129, 0.247] | <0.001 |
| Birth order | -0.029 | [-0.041, -0.017] | <0.001 |
| Residence | 0.245 | [0.097, 0.393] | 0.001 |
| Child age | 0.013 | [0.007, 0.018] | <0.001 |
| Region: Tigray (Reference) |  |  |  |
| Afar | -0.108 | [-0.292, 0.075] | 0.246 |
| Amhara | 0.215 | [0.091, 0.339] | 0.001 |
| Oromia | 0.307 | [0.188, 0.426] | <0.001 |
| Somali | 0.184 | [0.006, 0.362] | 0.043 |
| Benishangul | 0.097 | [-0.048, 0.242] | 0.191 |
| SNNPR | 0.348 | [0.229, 0.467] | <0.001 |
| Gambela | 0.020 | [-0.138, 0.178] | 0.802 |
| Harari | 0.312 | [0.157, 0.467] | <0.001 |
| Addis Ababa | 0.293 | [0.114, 0.472] | 0.001 |
| Dire Dawa | -0.142 | [-0.292, 0.007] | 0.062 |
| Year : 2000 (Reference) |  |  |  |
| 2005 | 0.170 | [0.028, 0.311] | 0.019 |
| 2011 | 0.013 | [-0.076, 0.101] | 0.781 |
| 2016 | 0.049 | [-0.045, 0.144] | 0.307 |

**Table 5S**. Full Pooled linear regression model for risk factors of WHZ in children age 24-59 months (n= 9129), 2000-2016

|  |  | 24-59 months |  |
| --- | --- | --- | --- |
|  |  | n=9,129 |  |
|  | B | 95% CI | P-value |
| Perceived low weight at birth | -0.281 | [-0.336, -0.226] | <0.001 |
| Recent diarrhea | -0.189 | [-0.259, -0.119] | <0.001 |
| Child received vitamin A supplements | 0.044 | [-0.007, 0.094] | 0.093 |
| Basic Water | 0.016 | [-0.046, 0.077] | 0.616 |
| Basic sanitation | 0.040 | [-0.055, 0.136] | 0.407 |
| Attended 4+ ANC visits | 0.078 | [0.018, 0.138] | 0.011 |
| Wealth score (0-10) | 0.055 | [0.031, 0.080] | <0.001 |
| Livelihood: Pastoral | -0.084 | [-0.222, 0.055] | 0.237 |
| Month of interview: Lean season | 0.156 | [0.006, 0.305] | 0.041 |
| Maternal education: Secondary/higher | 0.153 | [0.054, 0.251] | 0.002 |
| Child sex | 0.090 | [0.042, 0.137] | <0.001 |
| Birth order | -0.006 | [-0.015, 0.003] | 0.21 |
| Residence | 0.046 | [-0.070, 0.163] | 0.435 |
| Child age | -0.001 | [-0.003, 0.002] | 0.499 |
| Region: Tigray (Reference) |  |  |  |
| Afar | 0.050 | [-0.097, 0.197] | 0.506 |
| Amhara | -0.022 | [-0.117, 0.073] | 0.648 |
| Oromia | 0.120 | [0.021, 0.220] | 0.018 |
| Somali | -0.352 | [-0.504, -0.200] | <0.001 |
| Benishangul | -0.113 | [-0.232, 0.007] | 0.064 |
| SNNPR | 0.248 | [0.148, 0.348] | <0.001 |
| Gambela | -0.027 | [-0.150, 0.096] | 0.67 |
| Harari | 0.114 | [-0.023, 0.251] | 0.103 |
| Addis Ababa | 0.322 | [0.176, 0.468] | <0.001 |
| Dire Dawa | -0.103 | [-0.238, 0.031] | 0.133 |
| Year : 2000 (Reference) |  |  |  |
| 2005 | 0.044 | [-0.090, 0.178] | 0.521 |
| 2011 | 0.013 | [-0.056, 0.082] | 0.719 |
| 2016 | 0.051 | [-0.023, 0.125] | 0.18 |

^1^Beta coefficients (95% CI) are estimated using linear regression with a robust variance estimator.

**Table 6S**. Full Pooled linear regression model to identify risk factors of WHZ in children age 0-59 months (n= 20,528), 2000-2016^1^

|  |  | 0-59 months |  |
| --- | --- | --- | --- |
|  |  | n=20,528 |  |
|  | B | 95% CI | P-value |
| Perceived low weight at birth | -0.284 | [-0.325, -0.242] | <0.001 |
| Recent diarrhea | -0.260 | [-0.308, -0.213] | <0.001 |
| Child received vitamin A supplements | -0.026 | [-0.066, 0.014] | 0.202 |
| Basic Water | -0.019 | [-0.065, 0.028] | 0.420 |
| Basic sanitation | 0.093 | [0.017, 0.169] | 0.019 |
| Attended 4+ ANC visits | 0.090 | [0.041, 0.139] | <0.001 |
| Wealth score (0-10) | 0.098 | [0.079, 0.116] | <0.001 |
| Livelihood: Pastoral | -0.113 | [-0.216, -0.009] | 0.033 |
| Month of interview: Lean season | 0.214 | [0.091, 0.336] | 0.001 |
| Maternal education: Secondary/higher | 0.151 | [0.073, 0.227] | <0.001 |
| Residence | 0.264 | [0.164, 0.364] | <0.001 |
| Child age | 0.003 | [0.001, 0.004] | <0.001 |
| Child sex | 0.122 | [0.085, 0.159] | <0.001 |
| Birth Order | -0.015 | [-0.022, -0.008] | <0.001 |
| Region: Tigray Ref |  |  |  |
| Afar | -0.095 | [-0.223, 0.034] | 0.148 |
| Amhara | 0.085 | [0.005, 0.165] | 0.036 |
| Oromia | 0.216 | [0.137, 0.294] | <0.001 |
| Somali | -0.109 | [-0.227, 0.010] | 0.072 |
| Benishangul | -0.011 | [-0.109, 0.087] | 0.828 |
| SNNPR | 0.274 | [0.193, 0.354] | <0.001 |
| Gambela | -0.031 | [-0.133, 0.071] | 0.549 |
| Harari | 0.141 | [0.037, 0.246] | 0.008 |
| Addis Ababa | 0.248 | [0.136, 0.360] | <0.001 |
| Dire Dawa | -0.187 | [-0.293, -0.082] | 0.001 |
| Year : Ref 2000 |  |  |  |
| 2005 | 0.058 | [-0.052, 0.167] | 0.3 |
| 2011 | -0.041 | [-0.100, 0.019] | 0.182 |
| 2016 | 0.001 | [-0.063, 0.065] | 0.976 |

^1^Beta coefficients (95% CI) are estimated using linear regression with a robust variance estimator.

**Table 7S**. Pooled linear regression analysis to identify risk factors of WHZ in children age 6-23 months (n= 6,154), 2005-2016^1^

|  | 6-23 months | | |
| --- | --- | --- | --- |
|  | B | 95% CI | P-value |
| Perceived low weight at birth | -0.334 | [-0.410,-0.259] | <0.001 |
| Recent diarrhea | -0.211 | [-0.292,-0.131] | <0.001 |
| Child fed five or more food groups (MDD-5) | 0.007 | [-0.108,0.123] | 0.900 |
| Child received vitamin A supplements | -0.005 | [-0.076,0.067] | 0.895 |
| Child has received age appropriate vaccination | 0.041 | [-0.041,0.124] | 0.325 |
| Basic Water | -0.068 | [-0.148,0.012] | 0.094 |
| Basic toilet facility | 0.182 | [0.056,0.307] | 0.005 |
| Safe disposal of child stools | 0.063 | [-0.016,0.143] | 0.118 |
| Attended 4+ ANC visits | 0.077 | [-0.004,0.158] | 0.063 |
| Wealth score (0-10) | 0.123 | [0.087,0.159] | <0.001 |
| Livelihood: Pastoral | 0.206 | [0.010,0.401] | 0.039 |
| Month of interview: Lean season | 0.247 | [0.068,0.426] | 0.007 |
| Maternal education: Secondary/higher | 0.125 | [-0.009,0.259] | 0.067 |

^1^Beta coefficients (95% CI) are estimated using linear regression with a robust variance estimator. Models were adjusted for child age, child sex, birth order, residence, region, and survey round.

**Table 8S.** Oaxaca-Blinder decomposition of driers of change in WHZ between 2000 and 2016 in children age 6-23 months

| WHZ | Coef. | 95% CI | P-value |
| --- | --- | --- | --- |
| Overall |  |  |  |
| Group 1 | -0.548 | [-0.639, -0.458] | <0.001 |
| Group 2 | -0.899 | [-0.980, -0.818] | <0.001 |
| Difference | 0.351 | [0.230, 0.473] | <0.001 |
| Explained | 0.164 | [0.063, 0.264] | 0.001 |
| unexplained | 0.187 | [0.024, 0.351] | 0.025 |
| Explained |  |  |  |
| Perceived low weight at birth | 0.025 | [0.010, 0.041] | 0.001 |
| Recent diarrhea | 0.052 | [0.027, 0.076] | <0.001 |
| Wealth score (0-10) | 0.085 | [0.019, 0.150] | 0.011 |
| unexplained |  |  |  |
| Perceived low weight at birth | 0.005 | [-0.060, 0.070] | 0.882 |
| Recent diarrhea | 0.064 | [0.001, 0.127] | 0.046 |
| Child received vitamin A supplements | -0.022 | [-0.125, 0.080] | 0.669 |
| Basic Water | 0.024 | [-0.044, 0.092] | 0.49 |
| Basic sanitation | -0.001 | [-0.006, 0.004] | 0.728 |
| Attended 4+ ANC visits | -0.005 | [-0.055, 0.045] | 0.844 |
| Residence | 0.271 | [-0.218, 0.761] | 0.277 |
| Livelihood: Pastoral | 0.002 | [-0.013, 0.018] | 0.761 |
| Month of interview: Lean season | 0.003 | [-0.001, 0.007] | 0.107 |
| Child sex | 0.011 | [-0.098, 0.121] | 0.838 |
| Maternal education: Secondary/higher | -0.007 | [-0.038, 0.024] | 0.661 |
| Age appropriate vaccination | -0.021 | [-0.092, 0.049] | 0.556 |
| Safe disposal of child stools | 0.010 | [-0.066, 0.086] | 0.795 |
| Wealth score (0-10) | 0.069 | [-0.117, 0.254] | 0.469 |
| Child age | 0.145 | [-0.134, 0.423] | 0.309 |
| Birth order | -0.053 | [-0.214, 0.108] | 0.52 |

**Table 9S.** Oaxaca-Blinder decomposition of driers of change in WHZ between 2000 and 2016 in children age 24-59 months

| WHZ | Coef. | 95% CI | P-value |
| --- | --- | --- | --- |
| Overall |  |  |  |
| Group 1 | -0.401 | [-0.472, -0.331] | <0.001 |
| Group 2 | -0.638 | [-0.701, -0.575] | <0.001 |
| Difference | 0.237 | [0.142, 0.331] | <0.001 |
| Explained | 0.105 | [0.031, 0.179] | 0.005 |
| unexplained | 0.131 | [0.020, 0.242] | 0.021 |
| Explained |  |  |  |
| Perceived low weight at birth | 0.022 | [0.009, 0.036] | 0.001 |
| Basic sanitation | 0.013 | [0.001, 0.026] | 0.034 |
| Wealth score (0-10) | 0.071 | [0.009, 0.132] | 0.024 |
| unexplained |  |  |  |
| Recent diarrhea | 0.008 | [-0.030, 0.046] | 0.696 |
| Perceived low weight at birth | -0.036 | [-0.095, 0.023] | 0.234 |
| Child received vitamin A supplements | 0.098 | [-0.005, 0.202] | 0.063 |
| Attended 4+ ANC visits | -0.019 | [-0.061, 0.023] | 0.384 |
| Basic Water | 0.001 | [-0.057, 0.058] | 0.977 |
| Basic sanitation | 0.003 | [0.000, 0.006] | 0.036 |
| Residence | -0.036 | [-0.438, 0.366] | 0.861 |
| Wealth score (0-10) | -0.094 | [-0.268, 0.080] | 0.289 |
| Maternal education: Secondary/higher | -0.005 | [-0.029, 0.019] | 0.691 |
| Livelihood: Pastoral | -0.005 | [-0.016, 0.006] | 0.407 |
| Month of interview: Lean season | -0.001 | [-0.003, 0.002] | 0.546 |
| Child age | -0.305 | [-0.623, 0.014] | 0.061 |
| Child sex | -0.063 | [-0.138, 0.012] | 0.099 |
| Birth order | 0.046 | [-0.099, 0.191] | 0.533 |

**Table 10S:** Month of data collection across EDHS rounds

| **Survey round** | **Month of data collection** | | | | | | | | | | | |
| --- | --- | --- | --- | --- | --- | --- | --- | --- | --- | --- | --- | --- |
|  | **Jan** | **Feb** | **Mar** | **Apr** | **May** | **Jun** | **Jul** | **Aug** | **Sep** | **Oct** | **Nov** | **Dec** |
| **EDHS 2000** |  |  |  |  |  |  |  |  |  |  |  |  |
| EDHS 2005 |  |  |  |  |  |  |  |  |  |  |  |  |
| EDHS 2011 |  |  |  |  |  |  |  |  |  |  |  |  |
| EDHS 2016 |  |  |  |  |  |  |  |  |  |  |  |  |
